# Deep Learning for the Pathologic Diagnosis of Hepatocellular Carcinoma, Cholangiocarcinoma, and Metastatic Colorectal Cancer

**DOI:** 10.3390/cancers15225389

**Published:** 2023-11-13

**Authors:** Hyun-Jong Jang, Jai-Hyang Go, Younghoon Kim, Sung Hak Lee

**Affiliations:** 1 Department of Physiology, CMC Institute for Basic Medical Science, College of Medicine, The Catholic University of Korea, Seoul 06591, Republic of Korea; hjjang@catholic.ac.kr; 2 Department of Pathology, Dankook University College of Medicine, Cheonan 31116, Republic of Korea; cyjy555@daum.net; 3 Department of Hospital Pathology, Seoul St. Mary’s Hospital, College of Medicine, The Catholic University of Korea, Seoul 06591, Republic of Korea; glabella85@gmail.com

**Keywords:** deep learning, pathology, hepatocellular carcinoma, cholangiocarcinoma, colorectal cancer

## Abstract

**Simple Summary:**

The pathologic diagnosis of primary and secondary liver cancers can often be difficult. Artificial intelligence (AI) presents potential solutions to these difficulties by aiding in the histopathological diagnosis of tumors using digital whole slide images (WSIs). We developed an AI diagnostic assistant using a deep learning model for distinguishing hepatocellular carcinoma, cholangiocarcinoma, and metastatic colorectal cancer using WSIs. Overall, the classifiers were highly accurate, showing significant potential for improving liver cancer diagnosis and advancing precision medicine. However, additional research is required to further refine and validate these promising tools.

**Abstract:**

Diagnosing primary liver cancers, particularly hepatocellular carcinoma (HCC) and cholangiocarcinoma (CC), is a challenging and labor-intensive process, even for experts, and secondary liver cancers further complicate the diagnosis. Artificial intelligence (AI) offers promising solutions to these diagnostic challenges by facilitating the histopathological classification of tumors using digital whole slide images (WSIs). This study aimed to develop a deep learning model for distinguishing HCC, CC, and metastatic colorectal cancer (mCRC) using histopathological images and to discuss its clinical implications. The WSIs from HCC, CC, and mCRC were used to train the classifiers. For normal/tumor classification, the areas under the curve (AUCs) were 0.989, 0.988, and 0.991 for HCC, CC, and mCRC, respectively. Using proper tumor tissues, the HCC/other cancer type classifier was trained to effectively distinguish HCC from CC and mCRC, with a concatenated AUC of 0.998. Subsequently, the CC/mCRC classifier differentiated CC from mCRC with a concatenated AUC of 0.995. However, testing on an external dataset revealed that the HCC/other cancer type classifier underperformed with an AUC of 0.745. After combining the original training datasets with external datasets and retraining, the classification drastically improved, all achieving AUCs of 1.000. Although these results are promising and offer crucial insights into liver cancer, further research is required for model refinement and validation.

## 1. Introduction

Primary liver cancer is the sixth-most common malignancy and the third leading cause of cancer-related deaths worldwide [1]. Hepatocellular carcinoma (HCC) and cholangiocarcinoma (CC) are the two primary types of liver malignancy. HCC is a malignant epithelial tumor that displays features of hepatocellular differentiation [2]. HCC has become one of the most common cancers worldwide, with a growing incidence rate over the past three decades [3]. While great achievements have been made in the histopathological classification of HCC, the diagnostic process is labor-intensive and requires pathologists to meticulously evaluate pathological images, with potential inter- and intra-observer variations [4,5]. In addition, significant intratumoral heterogeneity in HCC tissues may present a challenge when attempting to analyze morphological features solely through visual examination [6]. Along with HCC, CC is the second-most common primary liver cancer, comprising 15% of primary hepatic malignancies [7]. CC is a malignant tumor that exhibits biliary epithelial features [8]. Most CCs are diagnosed histopathologically as adenocarcinomas.

The diagnostic distinction between HCC and CC is of significant importance in terms of therapeutic implications. For instance, although orthotopic liver transplantation is widely accepted as a therapeutic approach for patients with HCC, it is contraindicated in patients with CC [9]. However, diagnostic differentiation between the two can occasionally be challenging, even for highly specialized pathologists [10,11].

Secondary liver cancer originates from a malignant tumor outside the liver that subsequently metastasizes. Secondary liver tumors predominantly consist of carcinomas, followed by melanoma, sarcoma, and lymphoma, in that order of frequency. The primary cancers that most frequently metastasize to the liver include colorectal, breast, lung, and gastric carcinomas [12]. About 20–30% of patients diagnosed with colorectal cancer (CRC) already have metastatic manifestations [13,14]. The liver is the most common site of CRC metastasis. When identifying liver metastases from CRC, the main differential diagnosis is CC, as most CRCs are predominantly adenocarcinomas [15].

Recently, advances in artificial intelligence (AI) have resulted in the development of highly efficient algorithms for various purposes in the medical field including pathology, with a subset of devices making their way into commercialization for clinics [16,17,18]. A representative task is the histopathological classification of tumors, which is essential for predicting outcomes and determining treatment [19,20]. More and more pathology laboratories are transitioning to the regular use of digital slides in the form of whole slide images (WSIs) for their daily diagnostic workflows [21,22,23]. The transformation from traditional microscopy to WSIs has facilitated the incorporation of AI support systems into pathology, thereby enhancing the efficiency, accuracy, and consistency. Consequently, this has enabled the emergence of cutting-edge techniques via deep learning (DL) [24,25,26,27]. Recent studies have confirmed the efficacy of AI in pathology for identifying tumors in several organs, including the lungs, stomach, and breasts [19,20,28,29,30]. However, the application of DL-based histopathological analysis for the diagnosis of HCC and CC has rarely been documented.

In this study, we aimed to develop a deep learning-based, fully automated model for the differential diagnosis of HCC, CC, and metastatic CRC (mCRC) using histopathological images. The potential utility of our results in the clinical setting is also discussed.

## 2. Materials and Methods

### 2.1. Datasets

Formalin-fixed paraffin-embedded (FFPE) slides of HCC and CC samples were obtained from The Cancer Genome Atlas (TCGA). After a basic quality review, 366 and 39 WSIs were selected for HCC (TCGA-LIHC) and CC (TCGA-CHOL), respectively. As the number of WSIs was too different between TCGA-LIHC and TCGA-CHOL to train a DL-based classifier, we collected 156 CC WSIs from Dankook University Hospital (DKUH-CHOL). Furthermore, 179 mCRC WSIs were collected from Dankook University Hospital (DKUH-META). For the external validation of the trained classifier, 31 and 29 WSIs of HCC and CC, respectively, were collected from Seoul St. Mary’s Hospital (SSMH dataset). All tissues included in this study were stained with hematoxylin and eosin (H&E). Patient characteristics and clinicopathological features of all cohorts are summarized in Table S1.

### 2.2. Deep Learning Models

**Figure 1:**
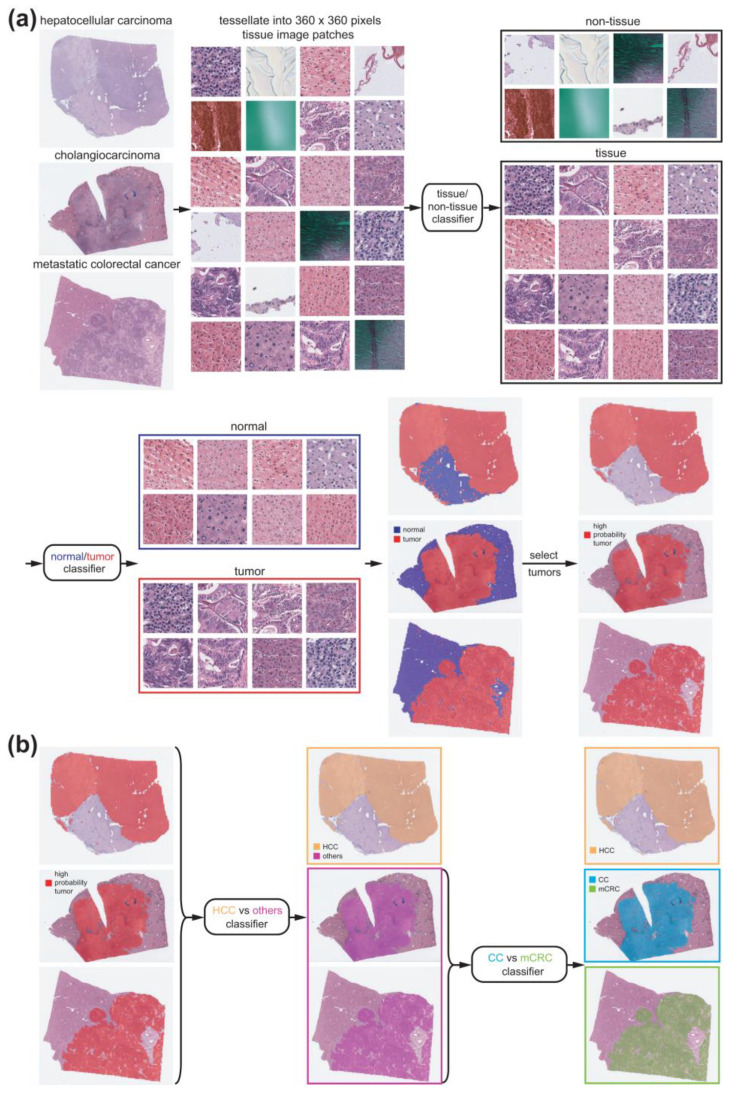
Procedure for the discrimination of different cancer types. (**a**) Tissue/non-tissue and normal/tumor classifiers are sequentially applied to separate proper tumor tissues from the whole slide images. (**b**) The first classifier discriminates hepatocellular carcinoma (HCC) from other cancer types. Then, the second classifier discriminates cholangiocarcinoma (CC) from metastatic colorectal cancer (mCRC).

Because the WSI was too large to be classified by a DL model as a whole, we trained the classifiers on small tissue image patches of 360 × 360 pixels at 20× magnification. To classify different cancer types, comparisons should only be made for cancer tissues. Therefore, cancer tissue image patches should be collected before classifying the cancer types. We sequentially applied two different DL-based tissue classifiers to collect cancer tissue image patches (Figure 1a).

**Figure 2:**
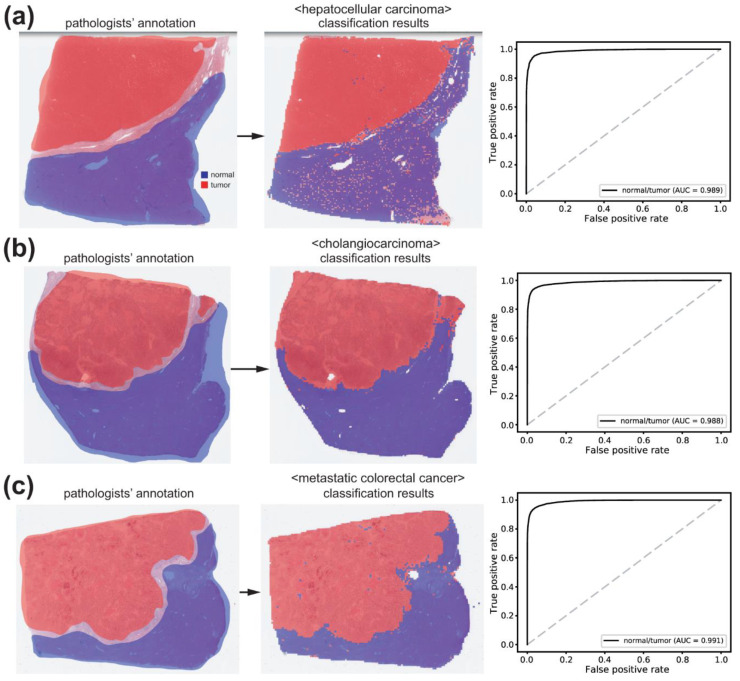
Results for normal/tumor classification. The patch-level classification results for hepatocellular carcinoma (**a**), cholangiocarcinoma (**b**), and metastatic colorectal cancer (**c**) were demonstrated with representative tissue images. Left panels: pathologists’ annotation. Middle panels: classification results by the normal/tumor classifier. Right panels: the receiver operating characteristic curves for the patch-level classification results. AUC: area under the curve.

First, proper tissue images in WSIs should be discriminated from multiple artifacts, including air bubbles, compression artifacts, out-of-focus blurring, pen markings, tissue folding, and white backgrounds. We reused a tissue/non-tissue classifier from our previous study to eliminate these artifacts [31]. A normal/tumor classifier was then trained to discriminate cancerous tissues from normal tissues. Three pathologists annotated the normal/tumor tissue regions in the WSIs of HCC, CC, and mCRC (Figure 2, left panels).

Normal and tumor tissue image patches were collected from all three cancer types to train a classifier that could discriminate normal/tumor tissues from the three cancer types simultaneously. We randomly selected 90% of normal and tumor tissue image patches to train the classifier and evaluated the performance of the classifier on the remaining 10%. We adopted three popular convolutional neural network (CNN) models to train the normal/tumor classifier: AlexNet, ResNet-50, and Inception-v3. The TensorFlow DL library (version 1.15) was used to train each DL model (http://tensorflow.org, accessed on 23 February 2023). Before training and testing the classifiers, the tissue images were color-normalized. Data augmentation techniques, including random horizontal/vertical flipping and random rotation by 90°, were applied to the tissue image patches during the training of the classifiers.

For the classification of cancer types, we collected tumor patches with a tumor probability higher than 0.9 to include patches with prominent tumor features. Since we trained the models based on slide-level diagnoses, all tumor tissue image patches from a given slide inherit the same label from the slide-level diagnosis. We implemented slide-level 5-fold cross-validation for cancer type classification. In this scheme, five different sets of sides were used for the training, validation, and testing with proportions of 60%, 20%, and 20%, respectively. The validation datasets were used to evaluate the performance of the classifiers during training. The classifiers with the highest performances for the validation datasets were then used for the testing datasets. We implemented a two-step approach to fully discriminate between HCC, CC, and mCRC. First, a classifier was trained to discriminate HCC from other cancer types (CC and mCRC) (Figure 1b, left). Another classifier was trained to discriminate between CC and mCRC (Figure 1b, right).

Six computer systems equipped with Intel Core i9-12900K (Intel Corporation, Santa Clara, CA, USA) processors and dual NVIDIA RTX 3090 GPUs (NVIDIA corporation, Santa Clara, CA, USA) were used to train and test the DL models.

### 2.3. Visualization and Statistics

The classification results were overlapped on the WSIs with color-coded heatmaps to visualize the distribution of the different tissue types. The averages of the patch classification results were used to obtain the slide-level classification results. To demonstrate the performance of each classifier, receiver operating characteristic (ROC) curves and areas under the curves (AUCs) for the ROC curves are presented. In the case of cancer-type classifiers, ROC curves for the folds with the lowest and highest AUCs and for the concatenated results of all five folds are provided for a more precise evaluation of the classification performance on the 5-fold cross-validated datasets. For the concatenated results of all five folds, 95% confidence intervals (CIs) are also presented. Accuracy, sensitivity, specificity, and F1 score were calculated with cutoff values yielding a maximal Youden index (sensitivity + specificity − 1).

To compare the ROC curves, the Venkatraman’s permutation test with 1000 iterations was applied [32]. Statistical significance was set at *p* < 0.05.

## 3. Results

### 3.1. Normal/Tumor Classification

Normal/tumor tissue image patches were collected based on annotations from pathologists. Tissue images from HCC, CC, and mCRC were mixed to train a single classifier for normal/tumor discrimination of all tissue types. AlexNet, ResNet-50, and Inception-v3 models were used to train the normal/tumor classifiers. Overall, Inception-v3 exhibited the best classification performance (Supplementary Table S2). Therefore, we adopted the Inception-v3 model for further analysis in the present study. The normal/tumor classification results obtained using the Inception-v3 model are presented in Figure 2. When the classifier was applied to the WSIs for HCC, CC, and mCRC in the test sets, the AUCs were 0.989, 0.988, and 0.991, respectively. Although the classification performance was better for mCRC than for HCC or CC (both *p* < 0.05, Venkatraman’s permutation test), we concluded that the classification performance of the classifier was sufficient for collecting tumor tissues from all cancer types based on a qualitative review of the classification results (Figure 2, middle panels).

### 3.2. Classification of HCC/Other Cancer Types

**Figure 3:**
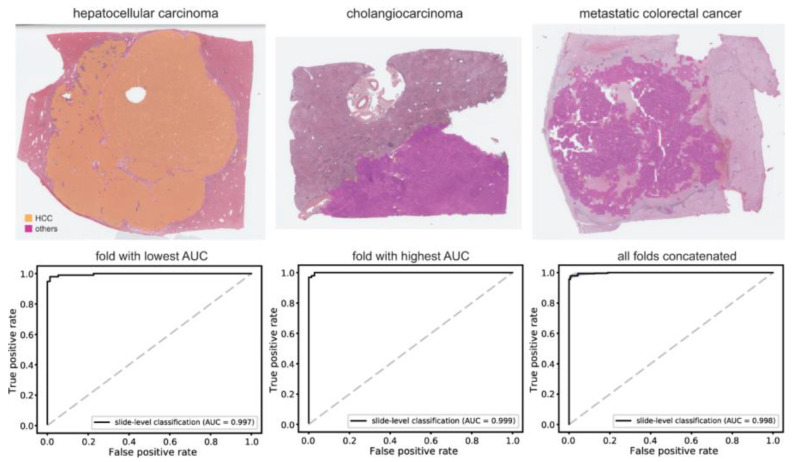
Classification results between hepatocellular carcinoma and other cancer types (cholangiocarcinoma and metastatic colorectal cancer). Upper panels: representative tissue images of hepatocellular carcinoma, cholangiocarcinoma, and metastatic colorectal cancers that were correctly classified by the classifier. Lower panels: the receiver operating characteristic curves of slide-level classification results for folds with the lowest and highest area under the curve (AUC) and concatenated results of all 5-folds. HCC: hepatocellular carcinoma.

Using a normal/tumor classifier, high-probability tumor tissues were collected to build a classifier to discriminate HCC from other cancer types (CC and mCRC). We adopted five-fold cross-validation for cancer type classification. Training was performed at least four times for each fold and the classifiers with the best AUC for the testing datasets were adopted to present the results in Figure 3. The average number of tissue image patches used for training the classifiers is summarized in Supplementary Table S3. As shown in the upper panels of Figure 3, most WSIs of HCC were clearly discriminated from those of CC and mCRC. The AUCs were 0.997 and 0.999 for fold-changes with the lowest and highest AUCs, respectively. The AUC for the concatenated results was 0.998 (95% CI, 0.997–0.999). The accuracy, sensitivity, specificity, and F1 score of the classifier are listed in Supplementary Table S4.

### 3.3. CC/mCRC Classification

**Figure 4:**
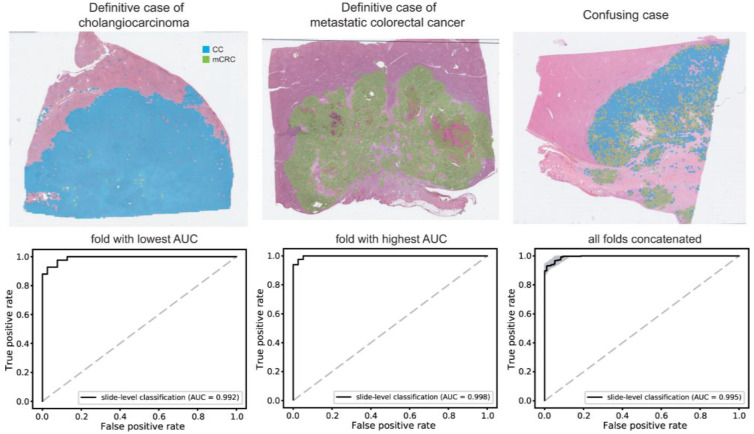
Classification results between cholangiocarcinoma and metastatic colorectal cancer. Upper panels: the representative whole slide images of clear cholangiocarcinoma, clear metastatic colorectal cancer, and confusing case with mixed classification results. Lower panels: the receiver operating characteristic curves of slide-level classification results for folds with the lowest and highest area under the curve (AUC) and concatenated results of all 5-folds. CC: cholangiocarcinoma, mCRC: metastatic colorectal cancer.

Subsequently, a classifier was developed to discriminate between CC and mCRC. The classification results are shown in Figure 4. Representative WSIs of clear CC, clear mCRC, and confusing cases with mixed classification results are shown in the upper panel. The AUCs were 0.992 and 0.998 for fold-changes with the lowest and highest AUCs, respectively. The AUC for the concatenated results was 0.995 (95% CI, 0.992–0.998). The accuracy, sensitivity, specificity, and F1 score of the classifier are listed in Supplementary Table S4.

### 3.4. Performance on an External Dataset

**Figure 5:**
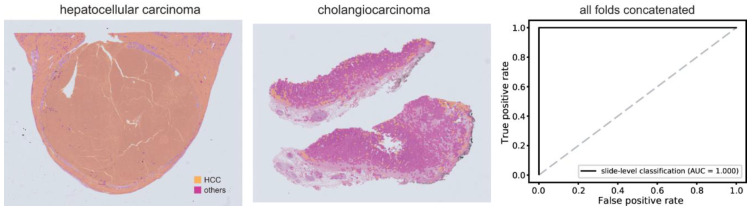
Classification results for the Seoul St. Mary’s Hospital (SSMH) dataset by a classifier trained with mixed datasets. Representative whole slide images of hepatocellular carcinoma (left) and cholangiocarcinoma (middle) from the SSMH dataset. The receiver operating characteristic curve of slide-level classification results for the concatenated results of all 5-folds is presented in the right panel. AUC: area under the curve, HCC: hepatocellular carcinoma.

To test whether the trained DL model performed well on an external dataset, we tested hepatocellular carcinoma/other cancer type classifiers on the SSMH dataset. The performance was poor with an AUC of 0.745, suggesting poor generalizability of the classifier to external datasets (Supplementary Figure S1). We split the SSMH dataset into five-folds and mixed the data with TCGA and DKUH data to retrain the classifier. When we retrained the classifier with mixed data from TCGA, DKUH, and SSMH tissue images, the classification results for the SSMH dataset significantly improved (Figure 5). The AUCs were all 1.000 for every fold, suggesting perfect classification. The classification performance of the new classifier on the original TCGA+DKUH datasets did not significantly improve despite the enlarged training datasets (*p* = 0.329 through Venkatraman’s permutation test, Supplementary Figure S2).

## 4. Discussion

In the present study, we adopted a two-step approach to discriminate between HCC, CC, and mCRC. Although DL-based classifiers can perform three classes of classification tasks directly, we split the task into two steps based on the dataset size. As summarized in Supplementary Table S3, more tissue imaging data are available for HCC. In general, DL-based classifiers do not perform well when the amount of training data is severely unbalanced [20]. As the data size can be balanced when CC and mCRC are mixed into a class, we first trained a classifier to discriminate between HCC and other cancer types. Furthermore, the second classifier for discriminating between CC and mCRC can also be trained on balanced datasets because the numbers of tissue images were similar for CC and mCRC. The overall AUCs for the two classifiers were 0.998 and 0.995, respectively. The higher AUC for the first classifier suggests that discriminating between HCC and other cancer types is a more obvious task than discriminating between CC and mCRC. Alternatively, it is a mere reflection of the larger training dataset sizes for the first classifier. We plan to collect more data for CC and mCRC to test whether the classification performance can be improved with more data.

While the discrimination performance showed promise, there were instances of misclassified cases and confusion due to mixed classification results, as illustrated in Figure 4. Enhancing the performance for challenging cases can be achieved through additional training methodologies, such as hard negative mining [33]. Additionally, a recent study has shown that a transformer network can enhance classification performance in comparison to a CNN [34]. These techniques hold the potential to enhance the performance of the HCC-CC-mCRC classifier for clinical applications.

The generalizability of DL-based classifiers is an important issue for the wide application of DL models. Unfortunately, our previous studies have shown that classifiers for the discrimination of various molecular traits from tissue images based on TCGA datasets did not perform well on Korean datasets [35,36,37,38]. In the present study, although we attempted to enhance the generalizability using color normalization and data augmentation techniques, the performance of the first classifier was poor on the SSMH dataset. Because the tissue image data of HCC came exclusively from the TCGA dataset for training the classifier, the results also indicate that the cancer-type classifiers trained on TCGA datasets show poor generalizability for Korean data. Overfitting on the training dataset can account for this lack of generalizability. Nevertheless, we made efforts to mitigate overfitting by implementing early stopping during training and utilizing validation datasets. Thus, the poor generalizability might not solely stem from overfitting. We expect that the poor generalizability originates mainly from differences in tissue characteristics. The quality of H&E-stained tissue slides can vary depending on tissue preparation and staining procedures, including fixation methods, cutting methods, dye concentration, and staining time [39]. Furthermore, differences in slide scanners and scanning settings can affect the quality of the scanned tissue slides. Finally, ethnic differences in the datasets may have resulted in poor generalizability. When we mixed the SSMH data into the original training data to train a new classifier, the SSMH dataset achieved a perfect AUC of 1.000. To enhance the generalizability of DL-based tissue classifiers, it is important that the classifier is exposed to a variety of data. Therefore, it is necessary to collect larger datasets from multiple institutes [40]. We are currently conducting a multi-center research project that collects and annotates the representative pathology WSIs for major cancers including liver and colorectal carcinomas (2021–2025, https://www.codipai.org/, accessed on 23 February 2023), and plan to implement a large-scale validation study using these cohorts in the near future.

The pathological evaluation of tissue samples is a key step in the differential diagnosis of HCC, CC, and mCRC. Nevertheless, even with histological analysis, distinguishing between HCC, CCA, and mCRC can be challenging. In most cases, HCC is suspected or directly identified on H&E-stained sections. HCC is characterized by the presence of sheets of polygonal cells with cytological atypia and architectural abnormalities such as thickened hepatic plates. In contrast, CC consists of cells that exhibit diverse structural arrangements, such as glandular and solid growth patterns. CCs induce a marked desmoplastic fibrotic reaction [8]. However, CCs do not always show identifiable tubular structures; rather, tumor cells often form solid sheets that mimic HCC. When metastasizing to the liver, mCRC also has morphological features similar to those of primary liver cancer and may display characteristics of desmoplasia or fibrotic changes [12].

For time-sensitive tasks such as histopathologic tumor classification, an initial diagnostic impression needs to be promptly established from the examination of routine H&E-stained slides. Several ancillary tests, including immunohistochemistry, fluorescence in situ hybridization, and molecular assays, depend on this preliminary determination. Therefore, our deep learning algorithm has the potential to be used as a screening tool for the diagnosis of HCC, CC, and mCRC. Moreover, apart from simply diagnosing CRC from pathology WSIs, the results of molecular testing such as *K-RAS*, *TP53*, and *BRAF* mutations, which are crucial for determining the treatment strategy for CRC patients, can be predicted directly from the H&E-stained tissue slides [41]. In addition, machine learning and AI algorithms can incorporate genomic data including gene expression profiling with WSI datasets to develop predictive models for cancer classification, potentially enhancing optimized treatment decisions [42,43].

Recently, the rapid growth of the internet of things (IoT) and cloud computing has tremendous prospects in the field of surgical pathology [44]. With the integration of DL technologies with IoT and cloud computing, it is possible to develop a portable AI-assisted pathology diagnosis platform and increase the accuracy of diagnosis including primary liver cancers by using it as an initial diagnostic tool.

Since the approval of WSIs as primary diagnostic materials [45], many pathology laboratories have adopted slide scanners. The era of digital pathology has enabled the accumulation of digital tissue image archives for training various DL models. The discrimination of cancer tissue subtypes is the most basic task in cancer diagnosis. Recently, DL has been widely used for tissue subtyping in various cancers [31,46,47,48,49]. In the present study, we successfully trained classifiers to discriminate between HCC, CC, and mCRC. Because inter- and intra-observer variability in pathologists’ diagnoses is an important issue that limits the reliability of pathologic reports [50], DL-based classifiers can be adopted to reduce errors in pathologists’ diagnostic decisions. However, low generalizability is an important hurdle in the adoption of DL-based assistant systems. We expect that the accumulation of digitized tissue data will eventually help to develop more generalized DL models and will help to improve diagnostic accuracy in the near future.

## 5. Conclusions

Our pipeline may provide significant information for patients with liver cancer and thus contribute to precision medicine. The findings of this study suggest that an AI-powered platform may be able to detect and properly classify liver cancer with high accuracy and efficiency, facilitating the application of screening tools for histopathologic diagnosis. Further large-scale studies are required to refine the models developed and validate our results.

## Data Availability

The TCGA data presented in this study are openly available in the GDC data portal (https://portal.gdc.cancer.gov/, accessed on 10 July 2023). Further information is available from the corresponding authors upon request.

## Supplementary Materials

The following supporting information can be downloaded at https://www.mdpi.com/article/10.3390/cancers15225389/s1, Figure S1: The receiver operating characteristic curve of slide-level classification results for the external datasets (Seoul St. Mary’s Hospital datasets); Figure S2: Classification results between hepatocellular carcinoma and other cancer types (cholangiocarcinoma and metastatic colorectal cancer), classified by a classifier trained with mixed datasets; Table S1. Baseline characteristics of all cohorts; Table S2: Performance of AlexNet, ResNet-50, and Inception-v3 models for the normal/tumor classification represented as area under the curves for the receiver operating characteristic curves; Table S3: The average number of patches in each training fold for the 5-fold cross-validation scheme; Table S4: Accuracy, sensitivity, specificity, and F1 score of the classification results.
