# Supplementary material for: Deep Learning for the Pathologic Diagnosis of Hepatocellular Carcinoma, Cholangiocarcinoma, and Metastatic Colorectal Cancer
Source: Cancers (Basel). 2023 Nov 13;15(22):5389. doi: 10.3390/cancers15225389 (PMC10670046; doi:10.3390/cancers15225389)
Supplement: Supplementary file 1 [file cancers-15-05389-s001.zip › Supplementary Figures.pdf]

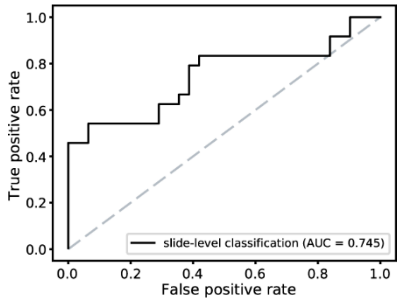

Supplementary Figure S1. The receiver operating characteristic curve of slide-level classification results for the external datasets (Seoul St. Mary's Hospital datasets). AUC: area under the curve.

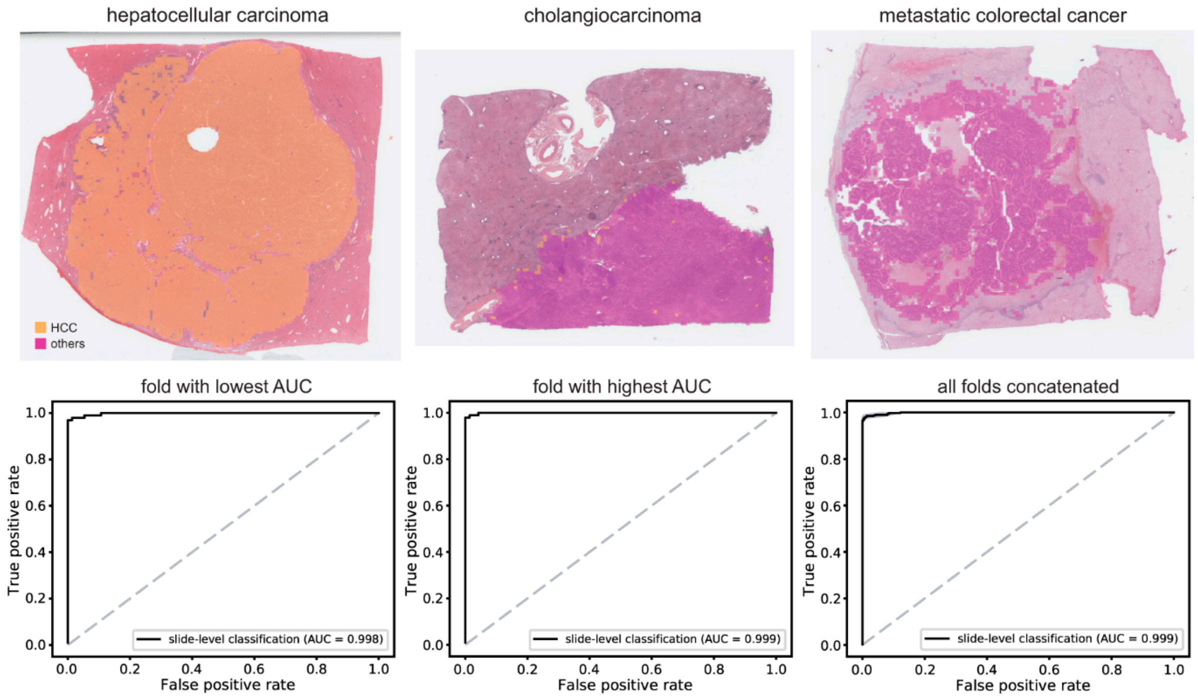

Supplementary Figure S2. Classification results between hepatocellular carcinoma and other cancer types (cholangiocarcinoma and metastatic colorectal cancer), classified by a classifier trained with mixed datasets. Upper panels: representative tissue images of hepatocellular carcinoma, cholangiocarcinoma, and metastatic colorectal cancers which were correctly classified by the classifier. Lower panels: the receiver operating characteristic curves of slide-level classification results for folds with the lowest and highest area under the curve (AUC) and concatenated results of all 5-folds. HCC: hepatocellular carcinoma.
