# Supplementary material for: Deep Learning for the Pathologic Diagnosis of Hepatocellular Carcinoma, Cholangiocarcinoma, and Metastatic Colorectal Cancer
Source: Cancers (Basel). 2023 Nov 13;15(22):5389. doi: 10.3390/cancers15225389 (PMC10670046; doi:10.3390/cancers15225389)
Supplement: Supplementary file 1 [file cancers-15-05389-s001.zip › Supplementary Tables.pdf]

Supplementary Table S1. Baseline characteristics of all cohorts

**TCGA-LIHC (N = 366)**	**Frequency (%)**
Age (years)	
Median	50.5 (16–85)
Sex	
M	248 (67.8)
F	118 (32.2)
Race	
White	185 (50.5)
Black	16 (4.4)
Asian	160 (43.7)
Other	2 (0.5)
Family History of Cancer	
No	211 (57.7)
Yes	105 (28.7)
pT stage	
1	182 (49.7)
2	91 (24.9)
3	79 (21.6)
4	12 (3.3)
pN stage	
0	255 (69.7)
1	4 (1.1)
Vascular Invasion	
Absent	207 (56.6)
Present (Microvascular)	93 (25.4)
Present (Macrovascular)	16 (4.4)
Surgical Procedure	
Segmentectomy	175 (47.8)
Lobectomy	161 (44.0)
Total Hepatectomy	1 (0.3)
Other	26 (7.1)
**TCGA-CHOL (N = 39)**	**Frequency (%)**
Age (years)	
Median	55.5 (29–82)
Sex	
M	16 (41.0)
F	23 (59.0)
Race	
White	35 (89.7)
Black	3 (7.7)
Asian	1 (2.6)
Family History of Cancer	
No	16 (41.0)
Yes	22 (56.4)
pT stage	
1	20 (51.3)
2	11 (28.2)
3	7 (17.9)
4	1 (2.6)
pN stage	
0	30 (76.9)
1	5 (12.8)
Vascular Invasion	
Absent	34 (87.2)
Present (Microvascular)	5 (12.8)

Surgical Procedure	
Segmentectomy	16 (41.0)
Lobectomy	16 (41.0)
Pancreaticoduodenectomy	3 (7.7)
Other	4 (10.3)
**DKUH-CHOL (N = 156)**	**Frequency (%)**
Age (years)	
Median	62 (38–86)
Sex	
M	98 (62.8)
F	58 (37.2)
Size (cm)	
Mean	4.82 (1.1–19.0)
pT stage	
1	40 (25.6)
2	73 (46.8)
3	23 (14.7)
4	16 (10.3)
pN stage	
0	51 (32.7)
1	50 (32.0)
Differentiation	
Well	26 (16.7)
Moderate	105 (67.3)
Poor	15 (9.6)
**DKUH-META (N = 179)**	**Frequency (%)**
Age (years)	
Median	59.5 (32–87)
Sex	
M	116 (64.8)
F	63 (35.2)
Size (cm)	
Mean	2.84 (0.2–13.0)
**SSMH-HCC (N = 31)**	**Frequency (%)**
Age (years)	
Median	63 (41–85)
Sex	
M	23 (74.2)
F	8 (25.8)
Size (cm)	
Mean	3.34 (1.2–12.0)
pT stage	
1	17 (54.8)
2	7 (22.6)
3	1 (3.2)
4	4 (12.9)
Vascular Invasion	
Absent	21 (67.7)
Present	10 (32.3)
Edmondson-Steiner grade	
I	1 (3.2)
II	11 (35.5)
III	13 (41.9)
IV	5 (16.1)
**SSMH-CC (N = 29)**	**Frequency (%)**
Age (years)	
Median	70.5 (45–85)

Sex	
M	17 (58.6)
F	12 (41.4)
Size (cm)	
Mean	3.7 (1.8–9.2)
pT stage	
1	10 (34.5)
2	14 (48.3)
3	3 (10.3)
4	1 (3.4)
pN stage	
0	11 (37.9)
1	11 (37.9)
Differentiation	
Well	5 (17.2)
Moderate	21 (72.4)
Poor	2 (6.9)

DKUH: Dankook University Hospital, SSMH: Seoul St. Mary's Hospital, TCGA: The Cancer Genome Atlas, HCC: Hepatocellular carcinoma, CC: Cholangiocarcinoma, META: metastatic colorectal cancer

Supplementary Table S2. Performance of AlexNet, ResNet-50, and Inception-v3 models for the normal/tumor classification represented as area under the curves for the receiver operating characteristic curves.

	hepatocellular carcinoma	cholangiocarcinoma	metastatic cancer
AlexNet	0.984	0.982	0.985
ResNet-50	0.989	0.986	0.989
Inception-v3	0.989	0.988	0.991

Supplementary Table S3. The average number of patches in each training fold for the 5-fold cross validation scheme. The average training epoch was also presented.

	Average number of hepatocellular carcinoma tissue patches for training	Average number of other cancer types tissue patches for training	Average training epoch
hepatocellular carcinoma/other cancer types classification (TCGA+DKUH datasets)	1,148,109	1,039,534	19
	Average number of cholangiocarcinoma tissue patches for training	Average number of metastatic cancer tissue patches for training	Average training epoch
cholangiocarcinoma / metastatic cancer classification (TCGA+DKUH datasets)	485,607	553,927	21
	Average number of hepatocellular carcinoma tissue patches for training	Average number of other cancer types tissue patches for training	Average training epoch
hepatocellular carcinoma/other cancer types classification (TCGA+DKUH+SSMH datasets)	1,527,854	1,472,698	17

DKUH: Dankook University Hospital, SSMH: Seoul St. Mary’s Hospital, TCGA: The Cancer Genome Atlas

Supplementary Table S4. Accuracy, sensitivity, specificity, and F1 score of the classification results. The measures were obtained with cutoff values yielding maximal Youden index (sensitivity + specificity - 1).

	Accuracy	Sensitivity	Specificity	F1 score
hepatocellular carcinoma/other cancer types classification (TCGA+DKUH datasets by a classifier trained with TCGA+DKUH datasets: Figure 3)	0.983	0.979	0.989	0.985
cholangiocarcinoma / metastatic cancer classification (TCGA+DKUH datasets by a classifier trained with TCGA+DKUH datasets: Figure 4)	0.946	0.925	0.990	0.959
hepatocellular carcinoma/other cancer types classification (SSMH datasets by a classifier trained with TCGA+DKUH datasets: Supplementary Figure S1)	0.745	0.417	1.000	0.588
hepatocellular carcinoma/other cancer types classification (SSMH datasets by a classifier trained with TCGA+DKUH +SSMH datasets: Figure 5)	1.000	1.000	1.000	1.000
hepatocellular carcinoma/other cancer types classification (TCGA+DKUH datasets by a classifier trained with TCGA+ DKUH+SSMH datasets: Supplementary Figure S2)	0.985	0.977	0.994	0.986

DKUH: Dankook University Hospital, SSMH: Seoul St. Mary’s Hospital, TCGA: The Cancer Genome Atlas
